# High sensitivity cardiac troponin and the under-diagnosis of myocardial infarction in women: prospective cohort study

**DOI:** 10.1136/bmj.g7873

**Published:** 2015-01-21

**Authors:** Anoop S V Shah, Megan Griffiths, Kuan Ken Lee, David A McAllister, Amanda L Hunter, Amy V Ferry, Anne Cruikshank, Alan Reid, Mary Stoddart, Fiona Strachan, Simon Walker, Paul O Collinson, Fred S Apple, Alasdair J Gray, Keith A A Fox, David E Newby, Nicholas L Mills

**Affiliations:** 1 BHF Centre for Cardiovascular Science, University of Edinburgh, Edinburgh EH16 4SB, UK; 2 Centre for Population Health Sciences, University of Edinburgh, UK; 3 Department of Biochemistry, Southern General Hospital, UK; 4 Department of Biochemistry, Royal Infirmary of Edinburgh, UK; 5 Department of Cardiology, St George’s Hospital and Medical School, UK; 6 Department of Laboratory Medicine and Pathology, University of Minnesota, USA; 7 Department of Emergency Medicine, Royal Infirmary of Edinburgh, UK

## Abstract

**Objective** To evaluate the diagnosis of myocardial infarction using a high sensitivity troponin I assay and sex specific diagnostic thresholds in men and women with suspected acute coronary syndrome.

**Design** Prospective cohort study.

**Setting** Regional cardiac centre, United Kingdom.

**Participants** Consecutive patients with suspected acute coronary syndrome (n=1126, 46% women). Two cardiologists independently adjudicated the diagnosis of myocardial infarction by using a high sensitivity troponin I assay with sex specific diagnostic thresholds (men 34 ng/L, women 16 ng/L) and compared with current practice where a contemporary assay (50 ng/L, single threshold) was used to guide care.

**Main outcome measure** Diagnosis of myocardial infarction.

**Results** The high sensitivity troponin I assay noticeably increased the diagnosis of myocardial infarction in women (from 11% to 22%; P<0.001) but had a minimal effect in men (from 19% to 21%, P=0.002). Women were less likely than men to be referred to a cardiologist or undergo coronary revascularisation (P<0.05 for both). At 12 months, women with undisclosed increases in troponin concentration (17-49 ng/L) and those with myocardial infarction (≥50 ng/L) had the highest rate of death or reinfarction compared with women without (≤16 ng/L) myocardial infarction (25%, 24%, and 4%, respectively; P<0.001).

**Conclusions** Although having little effect in men, a high sensitivity troponin assay with sex specific diagnostic thresholds may double the diagnosis of myocardial infarction in women and identify those at high risk of reinfarction and death. Whether use of sex specific diagnostic thresholds will improve outcomes and tackle inequalities in the treatment of women with suspected acute coronary syndrome requires urgent attention.

## Introduction

Major inequalities exist between men and women in the treatment and outcome of acute coronary syndromes, with an increase in both early and late deaths in women.^1^ ^2^ ^3^ Importantly, women with suspected acute coronary syndrome are less likely to have a diagnosis of myocardial infarction, an observation previously attributed to atypical symptoms and less reliable findings on electrocardiography.^4^ ^5^

The universal definition of myocardial infarction advocates the use of cardiac troponin assays, with an increase above the upper reference limit (derived from the 99th centile of a normal reference population) diagnostic of myocardial infarction in patients with symptoms or signs of myocardial ischaemia.^6^ Although the universal definition of myocardial infarction advocates the 99th centile as the upper reference limit, only one in three clinical laboratories currently use this as the diagnostic threshold, given the analytical imprecision of established troponin assays.^7^ More sensitive troponin assays improve the diagnosis of myocardial infarction,^8^ and use of these assays has been associated with reductions in recurrent myocardial infarction and death.^9^ ^10^

One of the major advances of high sensitivity assays over contemporary assays is that troponin concentrations can be quantified in most healthy people. These assays have identified potentially important differences between the sexes, with the 99th centile (upper reference limit) being twofold higher in men than in women.^11^ ^12^ Whether the use of a single diagnostic threshold with contemporary assays has contributed to the under-diagnosis of myocardial infarction in women is unknown.

We evaluated the effect of a high sensitivity assay for cardiac troponin I using sex specific diagnostic thresholds on the incidence of myocardial infarction. Using these new thresholds, we also compared the clinical characteristics and outcomes of patients with a diagnosis of myocardial infarction.

## Methods

### Study population

We prospectively identified consecutive patients presenting to the Royal Infirmary of Edinburgh, United Kingdom, from 1 August to 31 October 2012, in whom the attending doctor suspected an acute coronary syndrome. Patients not resident in the south east of Scotland were excluded from the study. We obtained information about the patients and their clinical outcomes through the TrakCare software application (InterSystems, Cambridge, MA) as previously described.^9^ ^10^

Serum troponin concentrations were measured on admission and repeated six or 12 hours after the onset of symptoms^13^ ^14^ using both a contemporary sensitive troponin I assay and a high sensitivity troponin I assay. Clinical decisions were based on the results of the contemporary assay only, with clinicians blinded to the results of the high sensitivity assay.

### Troponin assays

The ARCHITECT_*STAT*_troponin I assay (Abbott Laboratories, Abbott Park, IL) is a contemporary sensitive assay that has been validated in our institution.^9^ ^10^ According to the manufacturer, the limit of detection is 10 ng/L and the upper reference limit (99th centile) of a normal reference population is 28 ng/L. Under local laboratory conditions the interassay coefficient of variation is less than 10% at 50 ng/L, and this concentration is used as the diagnostic threshold. The diagnostic threshold (50 ng/L) for the contemporary assay was based on a standard assessment of precision using pooled serum at a range of low concentrations across multiple platforms and reagent lots. This was the lowest concentration that consistently provided a coefficient of variation of 10% or less.^9^ ^10^

The ARCHITECT_*STAT*_high sensitive troponin I assay (Abbott Laboratories, Abbott Park, IL) has a limit of detection of 1.2 ng/L and an interassay coefficient of variation of less than 10% at 4.7 ng/L. The upper reference limit (99th centile) based on 4590 samples from healthy men and women was determined by the manufacturer as 26 ng/L, and was twofold higher in men (34 ng/L) than in women (16 ng/L; see supplementary figure S1). These upper reference limits are consistent with other studies involving different ethnic groups.^12^ ^15^ An assessment of assay precision was performed locally, with our laboratories consistently able to achieve a coefficient of variation of less than 10% at 6 ng/L in pooled serum.^16^

### Diagnosis of myocardial infarction

The initial adjudication was undertaken using the peak troponin concentration from the contemporary assay while blinded to the high sensitivity troponin assay (fig 1). Where serial samples were available, we used peak and change in troponin concentration. Two cardiologists (ASVS, ALH) independently reviewed all clinical information, including non-invasive and invasive investigations and outcomes from admission to 30 days. Patients were classified as having type 1 myocardial infarction, type 2 myocardial infarction, myocardial injury, or unstable angina.^6^ ^17^ We defined type 1 myocardial infarction as myocardial necrosis in the context of an isolated presentation with suspected acute coronary syndrome with chest pain or evidence of myocardial ischaemia on the electrocardiogram. Patients with symptoms or signs of myocardial ischaemia as a result of increased oxygen demand or decreased supply (for example, tachyarrhythmia, hypotension, or anaemia) and myocardial necrosis were classified as having type 2 myocardial infarction. Myocardial injury was defined as evidence of myocardial necrosis in the absence of any clinical features of myocardial ischaemia.^6^ Unstable angina was defined as symptoms or signs of myocardial ischaemia in the absence of myocardial necrosis with evidence of myocardial ischaemia on resting electrocardiography or stress testing, or obstructive coronary artery disease on coronary angiography, or where the patient had a recurrent myocardial infarction or died within 30 days. Any discrepancies in adjudication were resolved by consensus between the two cardiologists through in-depth review of source documents. They then reclassified patients using troponin concentrations from the high sensitivity assay with both a single generic threshold (26 ng/L) and sex specific thresholds (34 ng/L for men and 16 ng/L for women).

**Figure fig1:**
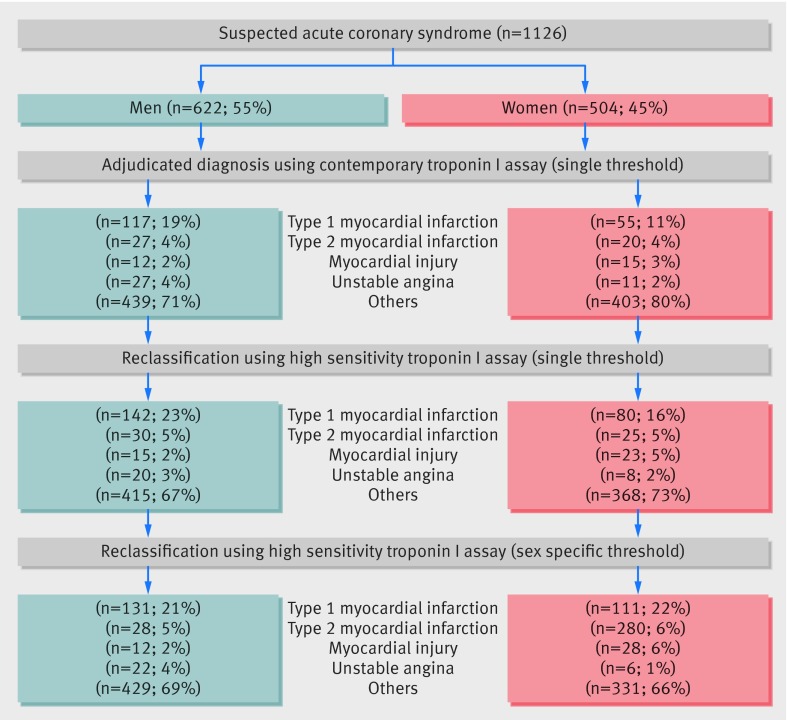
**Fig 1** Steps involved in adjudication and classification of patients with suspected acute coronary syndrome. Patients were adjudicated using the contemporary troponin I assay with a single threshold (50 ng/L) and then reclassified using the high sensitivity troponin I assay, with both single (26 ng/L) and sex specific (men 34 ng/L and women 16 ng/L) thresholds

### Follow-up

Subsequent readmission with myocardial infarction or death from any cause was recorded at 12 months. We used regional and national registries to ensure that follow-up was complete for the entire study population. TrakCare software application (InterSystems, Cambridge, MA) is a regional electronic patient record system, which provides data on all hospital admissions to both tertiary and secondary care hospitals in south east Scotland. Through requests for data from the National Records of Scotland we identified deaths occurring anywhere in the United Kingdom. We used these two sources to record all admissions to secondary and tertiary hospitals in our region and all deaths in the United Kingdom.^18^

### Statistical analysis

We compared continuous variables using Students *t* and Mann Whitney U tests as appropriate. For categorical data we used the χ^2^ test for between group comparisons and McNemar’s test for within group comparisons. The kappa statistic was used to assess agreement for the adjudicated diagnosis between the two independent adjudicators and was excellent (κ=0.97, 95% confidence interval 0.96 to 0.98).

Comparing diagnostic accuracy between two troponin assays in the absence of an independent reference standard is methodologically challenging.^19^ We used a modification of the method recommended by Glasziou and colleagues.^19^ This involves comparing four groups classified as normal (no myocardial infarction) or diseased (myocardial infarction) based on the contemporary sensitive and high sensitivity assay (respectively, normal/normal—group 1, disease/disease—group 3, normal/disease—group 2, and disease/normal—group 4) with the particular focus on comparing those with discrepant classifications (normal/disease—group 2 and disease/normal—group 4).^19^ For this comparison, a variable that is related to the condition, however imperfectly, but is independent of the assay result (conditional on the true unobserved disease state) is recommended. For example, evidence of myocardial ischaemia on electrocardiography could be used as such an “umpire test” wherein the discrepant group with the highest prevalence of ischaemia on electrocardiography would indicate the superior test. Consequently we compared a range of risk factor, clinical and outcome variables for all groups, which are likely to be independent of the assay under consideration but are conditional on the true disease state. To make a global comparison for these variables, we performed a weighted summation of the selected variables for all patients in each group, where the weighting of each variable was determined by its association with the disease/disease group. We generated logistic regression models of myocardial infarction (group 3 versus group 1, excluding group 2 and group 4 patients) on baseline clinical characteristics and outcomes (age, hypertension, hyperlipidaemia, diabetes, vascular disease, previous coronary revascularisation, presentation with chest pain, smoking status, electrocardiographic findings on admission, drugs on admission, Killip class, and outcomes at 12 months). To calculate the probability of myocardial infarction (disease/disease) for patients in all groups we used a parsimonious model (derived through backward selection). We used kernel density estimates to plot the frequency distribution of these estimates for each group.^20^

We modelled death or recurrent myocardial infarction using logistic regression with adjustment for age, renal function, and comorbidity, where group 1 (normal/normal) was the referent. Analyses were performed in SPSS Version 20.0.0 (Armonk, NY: IBM, USA, 2012) and R Version 3.0.0.

## Results

A total of 1126 consecutive patients with suspected acute coronary syndrome (mean age 66 years (SD 16 years), 504 (45%) women and 622 (55%) men) were identified (table 1).

**Table 1 tbl1:** Baseline characteristics of men and women with suspected acute coronary syndrome. Values are numbers (percentages) unless stated otherwise

Characteristics	Suspected acute coronary syndrome				Acute coronary syndrome*		
	All (n=1126)	Women (n=504)	Men (n=622)		Women (n=117)	Men (n=153)	P value†
Mean (SD) age (years)	66 (16)	69 (16)	63 (15)		75 (13)	67 (14)	<0.001
Chest pain	936 (83)	405 (81)	531 (85)		96 (82)	130 (86)	0.441
Risk factors:							
Smoker	277 (27)	79 (17)	198 (34)		20 (19)	47 (33)	0.012
Hypertension	596 (53)	271 (54)	325 (52)		71 (61)	83 (55)	0.348
Hyperlipidaemia	503 (45)	210 (42)	293 (47)		53 (45)	80 (53)	0.212
Diabetes mellitus	184 (16)	72 (14)	112 (18)		22 (19)	33 (22)	0.540
Medical history:							
Ischaemic heart disease	466 (42)	193 (38)	273 (44)		56 (48)	81 (54)	0.348
Myocardial infarction	292 (26)	105 (21)	187 (30)		37 (32)	55 (36)	0.412
Ischaemic stroke	93 (8)	39 (8)	54 (9)		13 (11)	13 (9)	0.493
Transient ischaemic attack	54 (5)	28 (6)	26 (4)		4 (3)	3 (2)	0.466
Peripheral vascular disease	27 (2)	5 (1)	22 (4)		3 (3)	10 (7)	0.157
Revascularisation:							
Previous PCI	166 (15)	50 (10)	116 (19)		14 (12)	33 (22)	0.033
Previous CABG	86 (8)	21 (4)	65 (10)		7 (6)	22 (15)	0.025
Haemodynamics:							
Mean (SD) systolic pressure (mm Hg)	135 (25)	138 (27)	133 (23)		138 (30)	131 (22)	0.057
Mean (SD) diastolic pressure (mm Hg)	73 (15)	71 (15)	73 (15)		73 (17)	73 (14)	0.816
Heart rate (beats/min)	80 (21)	81 (22)	79 (20)		83 (19)	79 (21)	0.226
Clinical chemistry:							
Mean (SD) haemoglobin (g/L)	134 (35)	128 (47)	139 (19)		126 (16)	138 (21)	<0.001
Mean (SD) creatinine (µmol/L)	87 (53)	79 (34)	93 (63)		91 (43)	103 (78)	0.153
Mean (SD) urea nitrogen (mmol/L)	7 (5)	7 (6)	6 (4)		8 (6)	7 (3)	0.119
Mean (SD) cholesterol (mmol/L)	4 (1)	5 (1)	5 (2)		5 (1)	5 (1)	0.115
Median (interquartile range) admission troponin I (ng/L)	6 (2-24)	6 (2-20)	7 (3-28)		29 (17-102)	69 (22-480)	0.025
Median (interquartile range) peak troponin I (ng/L)	7 (3-34)	6 (2-27)	8 (3-53)		50 (24-2630)	1230 (82-14 700)	<0.001
Electrocardiography:							
ST elevation	118 (11)	35 (7)	83 (14)		22 (19)	47 (31)	0.024
ST depression	106 (10)	59 (12)	47 (8)		24 (21)	32 (21)	0.920
T wave inversion	221 (20)	100 (21)	121 (20)		28 (24)	40 (26)	0.662
Drugs on admission:							
Aspirin	432 (39)	180 (36)	252 (41)		53 (46)	71 (48)	0.801
Clopidogrel	133 (12)	57 (11)	76 (13)		14 (12)	23 (15)	0.466
β blockers	319 (29)	143 (29)	176 (29)		40 (35)	52 (35)	0.984
ACE inhibitors	366 (33)	159 (32)	207 (34)		47 (41)	48 (32)	0.158
Statins	469 (42)	196 (39)	273 (45)		55 (48)	76 (51)	0.608

PCI=percutaneous coronary intervention; CABG=coronary artery bypass grafting; ACE=angiotensin converting enzyme.

*Baseline characteristics are provided for all patients with acute coronary syndrome (type 1 myocardial infarction or unstable angina) following reclassification using the high sensitivity assay with sex specific diagnostic thresholds.

†Comparison between men and women with diagnosis of acute coronary syndrome.

Women with acute coronary syndrome were older than men with acute coronary syndrome (75 (SD 13) years *v* 67 (SD 14) years), had similar cardiovascular risk factors but were at higher risk of mortality (GRACE score 130 (SD 32) *v *114 (SD 30), P<0.001)^21^ and less likely to have previously undergone coronary revascularisation. Women were as likely as men to present with chest pain (82% *v* 86%, P=0.441) and to have ST segment depression and T wave inversion on electrocardiograms but less likely to present with ST segment elevation (19% *v* 31%, P=0.024). Compared with men, women on admission had lower serum troponin concentrations (29 ng/L (interquartile range 17-102) *v* 69 (22-480) ng/L, P=0.025) and peak troponin concentrations (50 (24-2630) ng/L *v* 1230 (82-14 700) ng/L, P<0.001).

### Diagnosis of myocardial infarction

Using the contemporary troponin I assay with a threshold of 50 ng/L, 55 women (11%) and 117 men (19%) were classified as having type 1 myocardial infarction (figs 1 and 2). Using a high sensitivity assay, the number of women classified as having type 1 myocardial infarction increased to 80 (16%) with the generic (26 ng/L) threshold and to 111 (22%) with the sex specific (women 16 ng/L) threshold (P<0.001, figs 1 and 2 and supplementary table S1). In contrast, in men the diagnosis of type 1 myocardial infarction using the high sensitivity assay increased only slightly to 142 (23%) with the generic (26 ng/L) threshold and to 131 (21%) with the sex specific (men 34 ng/L) threshold (P<0.021).

**Figure fig2:**
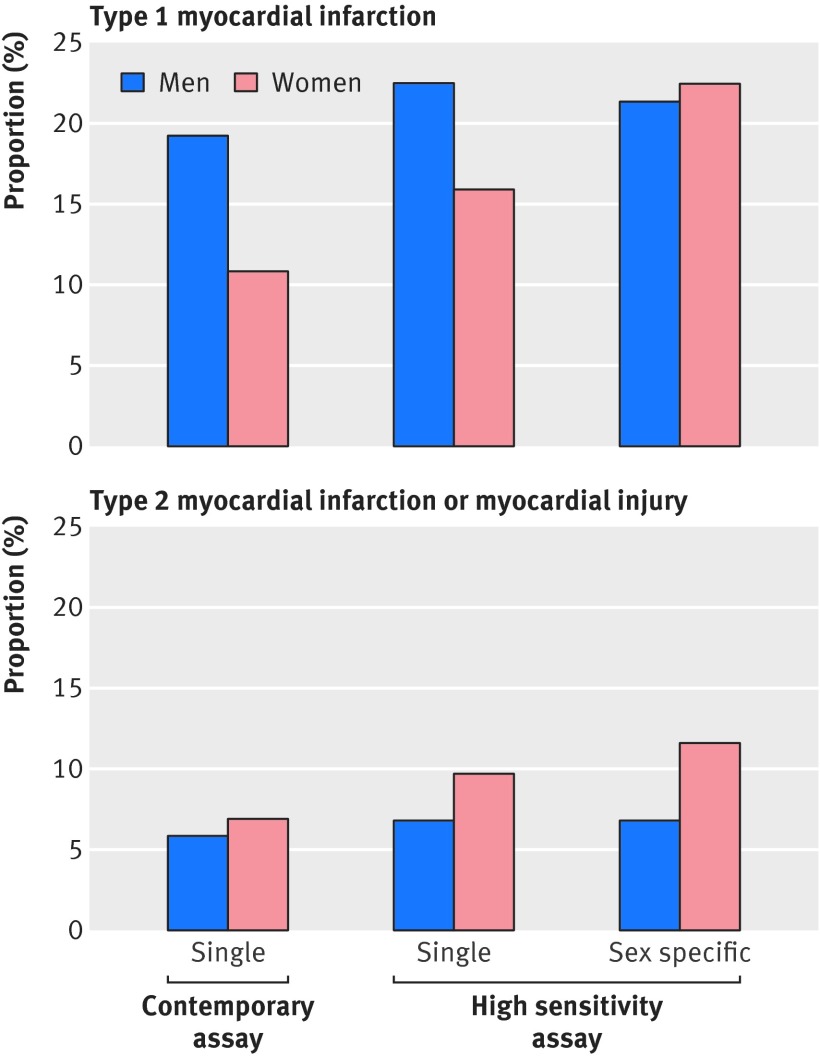
**Fig 2** Proportion of men and women with diagnosis of type 1 myocardial infarction and type 2 myocardial infarction or myocardial injury using the contemporary troponin I assay (single threshold 50 ng/L) and high sensitivity troponin I assay (single threshold 26 ng/L, and sex specific threshold 34 ng/L for men and 16 ng/L for women)

Overall, 20 women (4%) and 27 men (4%) received an adjudicated diagnosis of type 2 myocardial infarction using the contemporary troponin I assay with a threshold of 50 ng/L, and a further 15 women (3%) and 12 men (2%) had a diagnosis of myocardial injury (figs 1 and 2). Use of the high sensitivity assay and sex specific thresholds resulted in a small but significant increase in the number of women with a diagnosis of type 2 myocardial infarction or myocardial injury.

### Diagnostic accuracy

Patients were stratified into groups using both assays: those with no myocardial infarction (group 1), those with myocardial infarction (group 3) where the assays were concordant, and those identified as having myocardial infarction only using the high sensitivity assay with sex specific thresholds (group 2, table 2). No patients were identified as having myocardial infarction by the contemporary assay but not the high sensitivity assay (group 4). Patients in groups 2 and 3 had similar clinical characteristics and changes on electrocardiograms but differed from those patients without myocardial infarction (group 1; see supplementary tables S2 and S3). Age, chest pain, known vascular disease, myocardial ischaemia on the electrocardiogram, creatinine concentration, and death or recurrent myocardial infarction at one year were independently associated with a diagnosis of myocardial infarction on the index admission (C statistic 0.77, 95% confidence interval 0.72 to 0.82, likelihood ratio statistic 50.6, df=6, P<0.001) based on patients in group 1 and group 3. When this model was used to estimate the probability of myocardial infarction in all groups, those with discordant assay results in group 2 were similar to patients in group 3 but differed from those in group 1 (fig 3).

**Figure fig3:**
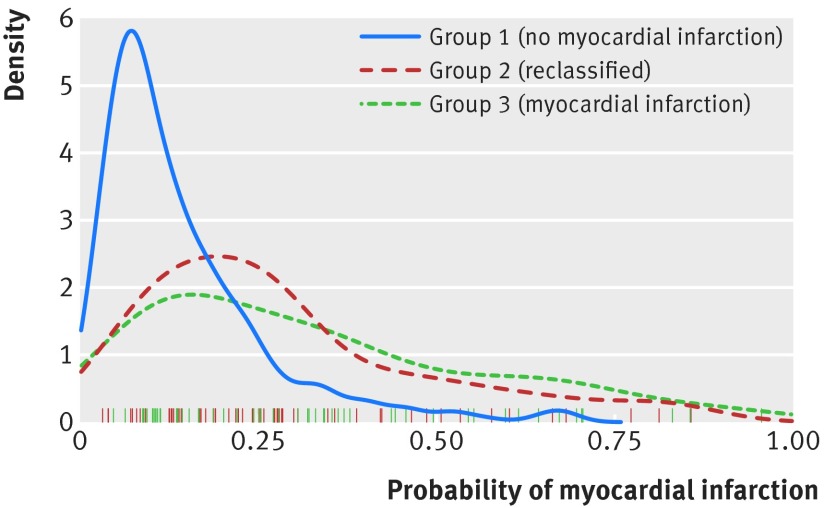
**Fig 3 **Density plots of predicted probabilities of myocardial infarction in women. Women were stratified into three groups using both assays: those with no myocardial infarction (group 1; solid blue line) and myocardial infarction (group 3; dashed green line) where the assays were concordant and those reclassified as having myocardial infarction using the high sensitivity assay with sex specific thresholds (group 2; dashed red line). Rug plot indicates the probability of myocardial infarction for individual patients in group 2 (red lines) and group 3 (green lines)

**Table 2 tbl2:** 2×2 tables comparing high sensitivity cardiac troponin assay (sex specific thresholds) with contemporary assay for diagnosis of type 1 myocardial infarction in men and women*

		Contemporary assay (single threshold)		
		Negative	Positive	Total
High sensitivity assay (sex specific threshold)	Women:			
	Negative	Group 1: 337	Group 4: 0	337
	Positive	Group 2: 56	Group 3: 55	111
	Total	393	55	448
	Men:			
	Negative	Group 1: 451	Group 4: 0	451
	Positive	Group 2: 14	Group 3: 117	131
	Total	465	117	582

*Patients were stratified into groups using both assays: those with no myocardial infarction (group 1) and myocardial infarction (group 3) where the assays were concordant and for those reclassified as having myocardial infarction using the high sensitivity assay with sex specific thresholds (group 2). There were no patients in whom troponin concentrations were below the threshold of the high sensitivity assay, but were above the threshold on the contemporary assay (group 4).

### Management of men and women with myocardial infarction

Women with a diagnosis of myocardial infarction (group 3) were less likely than men with myocardial infarction to be referred to a cardiologist (80% *v* 95%, P=0.004), undergo coronary angiography (47% *v* 74%, P=0.001) or percutaneous coronary revascularisation (29% *v *64%, P<0.001), or be prescribed statin treatment on discharge (60% *v* 85%, P=0.001; table 3). Differences persisted in our sensitivity analyses stratified by age and restricted to patients with non-ST segment elevation myocardial infarction (data not shown). Of those patients who underwent coronary angiography, the proportion of women and men with normal coronary arteries (14% *v* 5%) and with single vessel (31% *v* 42%), two vessel (14% *v* 23%), and three vessel (42% *v *29%) disease did not differ significantly (P=0.123 overall). Women identified as having myocardial infarction using only the high sensitivity assay with sex specific thresholds (group 2) were least likely to undergo investigations and treatment for myocardial infarction.

**Table 3 tbl3:** Management and outcome of patients with and without myocardial infarction.† Values are numbers (percentages) unless stated otherwise

Variables	Women					Men			
	Group 1: no myocardial infarction (n=337)	Group 2: reclassified (n=56)	Group 3: myocardial infarction (n=55)	P value		Group 1: no myocardial infarction (n=451)	Group 2: reclassified (n=14)	Group 3: myocardial infarction (n=117)	P value
Investigations:									
Cardiology referral	51 (15)	11 (20)	43 (80)	<0.001		84 (19)	5 (36)	111 (95)*	<0.001
Exercise tolerance test	6 (2)	1 (2)	0 (0)	0.606		16 (4)	0 (0)	1 (1)	0.233
Echocardiography	16 (5)	5 (9)	25 (46)	<0.001		20 (4)	0 (0)	38 (32)	<0.001
Coronary angiography	10 (3)	1 (2)	26 (47)	<0.001		21 (5)	3 (21)*	87 (74)*	<0.001
Revascularisation:									
PCI	5 (2)	0 (0)	16 (29)	<0.001		11 (2)	3 (21)*	75 (64)*	<0.001
Drugs on discharge:									
Aspirin	117 (35)	29 (57)	44 (85)	<0.001		185 (42)	10 (71)	95 (85)	<0.001
Clopidogrel	41 (12)	9 (18)	39 (75)	<0.001		62 (14)	8 (57)*	91 (81)	<0.001
β blockers	81 (24)	23 (45)	25 (48)	<0.001		130 (29)	9 (64)	61 (54)	<0.001
ACE inhibitors	90 (27)	19 (37)	31 (60)	<0.001		153 (35)*	5 (36)	81 (72)	<0.001
Statins	130 (39)	30 (59)	31 (60)	0.001		196 (45)	9 (64)	95 (85)*	<0.001
Outcome at 12 months									
Reinfarction or death	12 (4)	14 (25)	13 (24)	<0.001‡		35 (8)	5 (36)	22 (19)	0.001‡

PCI=percutaneous coronary intervention; ACE=angiotensin converting enzyme.

Patients with increased troponin concentrations classified as either type 2 myocardial infarction or myocardial injury are not included.

*P<0.05 (comparing men and women).

†Men and women were stratified into three groups using both assays: those with no myocardial infarction (group 1) and myocardial infarction (group 3) where the assays were concordant and for those reclassified as having myocardial infarction using the high sensitivity assay with sex specific thresholds (group 2).

‡Derived from log rank test.

### Clinical outcomes of men and women with myocardial infarction

Women with myocardial infarction identified using the high sensitivity assay with sex specific thresholds (group 2) or using both assays (group 3) had the highest risk of death or recurrent myocardial infarction at 12 months compared with those without myocardial infarction (group 1): 25% *v* 24% *v* 4%, respectively (P<0.001; table 3 and fig 4). Similar patterns were observed for men in groups 2 and 3 compared with men in group 1: 36% *v* 19% *v* 8%, respectively (P<0.001; table 3 and fig 4). After adjustment for age, renal function, and history of diabetes, women in groups 2 and 3 were at increased risk of death or recurrent myocardial infarction compared with women in group 1 (odds ratio 6.0, 95% confidence interval 2.5 to 14.4 and 5.8, 2.3 to 14.2, respectively, P<0.001 for both). Similarly, men in groups 2 and 3 were at increased risk of death or recurrent myocardial infarction compared with men in group 1 (5.3, 1.5 to 19.9, P=0.012, and 2.1, 1.1 to 3.8, P=0.021, respectively).

**Figure fig4:**
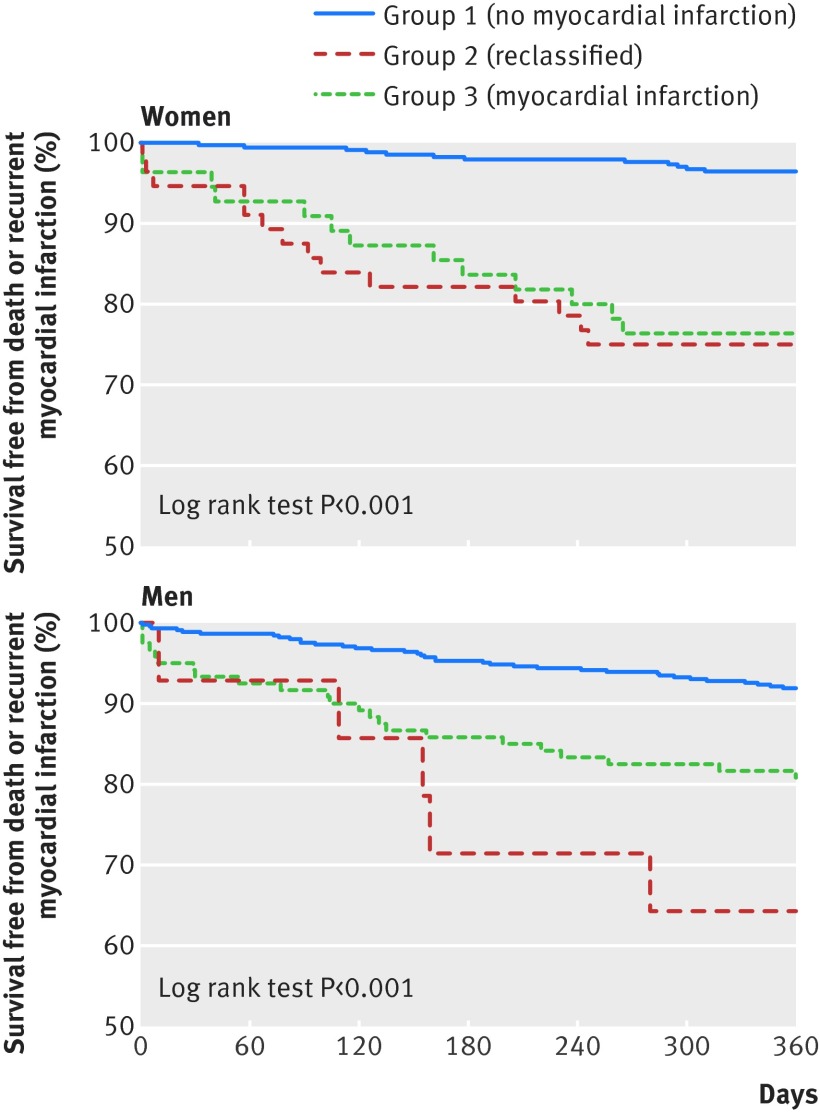
**Fig 4** Survival free from death or recurrent myocardial infarction in women and men with suspected acute coronary syndrome. Outcomes are shown for women and men with no myocardial infarction (group 1, solid blue line) and with myocardial infarction (group 3, dashed green line) where both assays were concordant, and for those reclassified as having myocardial infarction using the high sensitivity assay with sex specific thresholds (group 2, dashed red line)

We further evaluated outcomes in women, comparing the generic threshold (26 ng/L) with the sex specific threshold (16 ng/L) and obtained similar results. The event rate in women with troponin concentrations of 17-26 ng/L was sixfold higher (23%) than those in women with troponin concentrations of 16 ng/L or less (4%) and similar to women with troponin concentrations of 27-49 ng/L (25%, P for trend <0.001), suggesting that the use of sex specific thresholds will correctly identify more women at increased risk of recurrent myocardial infarction or death than a generic threshold.

## Discussion

Major differences exist in the diagnosis and management of myocardial infarction in men and women presenting with suspected acute coronary syndrome. Our findings using a high sensitivity troponin assay suggest that the use of contemporary assays with a single diagnostic threshold disproportionately under-diagnose myocardial infarction in women and contribute to sex inequalities in treatments and outcomes.

We make several novel and important observations. Firstly, we currently diagnose twice as many men with myocardial infarction as we do women. In patients with suspected acute coronary syndrome, use of a high sensitivity troponin assay with sex specific diagnostic thresholds doubles the diagnosis of myocardial infarction in women, such that the proportion of men and women with a diagnosis of myocardial infarction is now similar. Secondly, women identified as having myocardial infarction using only the high sensitivity assay with sex specific diagnostic thresholds had similar clinical characteristics and prognosis to those women diagnosed as having myocardial infarction using the contemporary assay. Thirdly, women are less likely than men to be referred to a cardiologist, to undergo coronary angiography or revascularisation, or to receive secondary prevention. These inequalities were most pronounced in those women with myocardial infarction who were not identified by the contemporary assay. Finally, women only identified using the high sensitivity assay with sex specific thresholds had the highest risk of death or recurrent myocardial infarction suggesting that these women have the greatest potential to benefit from reclassification and provision of treatment for acute myocardial infarction.

In patients with suspected acute coronary syndrome, why should the proportion of men and women diagnosed as having myocardial infarction differ? Men were twice as likely as women to have a diagnosis of myocardial infarction using a single threshold with our contemporary assay. This sex difference has been observed in other major clinical trials.^8^ ^22^ Historically this apparent under-diagnosis has been attributed to differences in presentation, with women more likely to have atypical symptoms and less reliable electrocardiographic changes.^5^ However, others^4^ and we have found that the same proportion of men and women reported chest pain and had electrocardiographic changes. Our findings suggest an additional explanation for the under-diagnosis of myocardial infarction in women: the use of inappropriate diagnostic thresholds. This then leads to the question of why women have lower troponin concentrations than men. Cardiac troponin concentrations correlate with left ventricular mass,^23^ ^24^ and women have less left ventricular mass than men.^25^ It is therefore not surprising that sex specific differences exist in the normal reference range for troponin and have been observed for other cardiac biomarkers, such as creatine kinase.^26^ However, to date contemporary assays have not had the sensitivity and precision to quantify troponin in more than 90% of healthy people and therefore were unable to determine sex specific upper reference limits.^12^ Our findings show that contemporary troponin assays with a single diagnostic threshold have disproportionately disadvantaged women and contributed to under-diagnosis of myocardial infarction.

The universal definition of myocardial infarction recognises that reference values may differ for high sensitivity troponin assays in men and women. These assays are widely available for clinical use outside the United States and are now recommended for the assessment of patients with suspected acute coronary syndrome by the National Institute for Health and Care Excellence in the United Kingdom.^27^ Importantly, although guidelines note that differences exist between the sexes in the reference range for troponin, no explicit recommendations on their use have been made owing to insufficient evidence. Our observations provide the first evaluation of the use of sex specific diagnostic thresholds for myocardial infarction. Although improvement in the diagnosis of type 1 myocardial infarction is likely to be beneficial, concerns have been raised about the potential for high sensitivity assays to reduce clinical specificity.^28^ ^29^ ^30^ In our cohort, secondary causes of myocardial injury and infarction were present in only a few patients but were more common in women than in men. Specificity was unchanged in men and only slightly lower in women suggesting that noticeable improvements in diagnostic sensitivity will not be offset by reductions in specificity.

We observed major sex differences in the management of myocardial infarction: less than half of women with an adjudicated diagnosis of myocardial infarction received optimal treatment compared with most men. Our findings are consistent with previous observations highlighting sex inequalities in the provision of healthcare to patients with myocardial infarction.^31^ ^32^ ^33^ While we believe that inappropriate single diagnostic thresholds contribute to these inequalities, differences in the age and clinical presentation of men and women may also be important. We therefore undertook sensitivity analyses stratified by age and ST segment elevation and found that inequalities persisted between men and women, suggesting that the use of inappropriate diagnostic thresholds is a major contributing factor.

Women are at higher short term and long term risk of death after acute coronary syndrome.^2^ ^32^ ^34^ ^35^ When a previous generation assay was used, women with suspected acute coronary syndrome were less likely to have increased plasma troponin concentrations,^5^ ^36^ and this may explain why clinicians were perhaps less likely to initiate evidence based treatments or to consider revascularisation. In our study, women with small undisclosed increases in troponin concentrations that were only discernible using the high sensitivity assay with sex specific diagnostic thresholds, were less likely to receive evidence based treatments and had the highest rates of reinfarction and death at 12 months. We have previously shown that lowering the diagnostic threshold of a contemporary assay for myocardial infarction increased the provision of treatment and was associated with a reduction in recurrent myocardial infarction and death.^9^ It is perhaps inevitable that further lowering the diagnostic threshold will increase the number of patients with a diagnosis of myocardial infarction. Whether implementation of high sensitivity troponin assays and use of sex specific diagnostic thresholds will improve outcomes through better targeting of treatments for coronary heart disease requires urgent attention and is the focus of an ongoing multicentre randomised controlled trial (clinical trials.gov NCT01852123).

### Limitations of this study

Our study has several limitations. Firstly, our cohort was derived from a single tertiary cardiac centre. However, we prospectively included all consecutive patients with no case selection and therefore believe our findings are both representative and generalisable. Secondly, we evaluated a single high sensitivity troponin I assay and diagnostic thresholds are likely to vary between different manufacturers and between troponin I and T assays.^12^ However, sex differences in the normal reference range have been observed across multiple troponin assays, including contemporary and high sensitivity methods.^11^ While only one in three laboratories currently uses the 99th centile for the diagnosis of myocardial infarction,^7^ we acknowledge that the impact of implementing high sensitivity assays with sex specific thresholds may be less noticeable in centres that use lower diagnostic thresholds based on manufacturers’ rather than local laboratory measures of assay precision. Thirdly, we accept that some patients with increased troponin concentrations may be misclassified as having type 1 myocardial infarction using the high sensitivity assay. This is because many patients with undetectable troponin concentrations using the contemporary assay were reassured and discharged without investigation such as coronary angiography or cardiac magnetic resonance imaging. However, our study reflects real world clinical practice and this approach to the diagnosis of myocardial infarction is consistent with major contemporary clinical trials,^37^ has been advocated in recent studies evaluating diagnostic accuracy of troponin assays,^8^ ^22^ and clearly identifies those patients at highest risk who are likely to benefit most from treatment for myocardial infarction.

### Conclusion

Coronary heart disease remains the leading cause of mortality in women. Our findings suggest that differences in outcome between men and women are only in part due to sex differences in the clinical presentation of coronary artery disease. We show that myocardial infarction is under-diagnosed in women and that this contributes to inequalities in the management and treatment of myocardial infarction.

**What is already known on this topic**

Major differences exist in the diagnosis, management, and outcomes of men and women with acute coronary syndromes
High sensitivity cardiac troponin assays have identified differences in the reference range for troponin between men and women
Single diagnostic thresholds may lead to under-diagnosis of myocardial infarction in women and contribute to sex inequalities in treatment and outcomes

**What this study adds**

Use of a high sensitivity cardiac troponin I assay with sex specific diagnostic thresholds may double the diagnosis of myocardial infarction in women, such that the proportion of men and women diagnosed as having myocardial infarction would be similar
Women with small undisclosed increases in troponin concentrations only discernible using the high sensitivity assay and sex specific diagnostic threshold, were less likely to receive evidence based treatments and had increased rates of recurrent myocardial infarction and death at 12 months
Whether implementation of high sensitivity troponin assays and use of sex specific diagnostic thresholds will improve outcomes through better targeting of treatments for coronary heart disease requires urgent attention

## Notes

We thank Ian King (Department of Clinical Biochemistry, Royal Infirmary of Edinburgh) and Shaoquing Du (Abbott Laboratories) for their assistance. This study is registered at clinicaltrials.gov (NCT01852123).

Contributors: ASVS and NLM conceived and designed the study. ASVS, MG, ALH, and KKL acquired the data. ASVS, DAM, NLM, and DEN analysed and interpreted the data. ASVS, DEN, and NLM drafted the initial manuscript. All authors critically revised the manuscript for important intellectual content. NLM had full access to all the data in the study and had final responsibility for the decision to submit for publication. All authors approved the final version of the report.

Ethical approval: This study was approved by the Scotland A Research Ethics Committee, NHS Lothian, Scotland, UK (12/SS/0115), in accordance with the Declaration of Helsinki.

Data sharing: No additional data available.

Transparency: The lead (NLM) affirms that this manuscript is an honest, accurate, and transparent account of the study being reported; that no important aspects of the study have been omitted; and that any discrepancies from the study as planned have been explained.

## Notes

Cite this as: *BMJ* 2015;350:g7873
