# High sensitivity cardiac troponin and the under-diagnosis of myocardial infarction in women: prospective cohort study

**DOI:** 10.1136/bmj.i4840

**Published:** 2016-09-06

This Research article (*BMJ* 2015;350:g7873, doi:10.1136/bmj.g7873) was mistakenly published under the CC BY NC licence initially online and in the March 2015 academic print issue, and should have been published under the CC BY licence. The online version has since been corrected.

