# Supplementary material for: High sensitivity cardiac troponin and the under-diagnosis of myocardial infarction in women: prospective cohort study
Source: BMJ. 2015 Jan 21;350:g7873. doi: 10.1136/bmj.g7873 (PMC4301191; doi:10.1136/bmj.g7873)
Supplement: Supplementary file 1 [file shaa020838.ww1_default.pdf]

DATA SUPPLEMENT

High-sensitivity cardiac troponin and the under diagnosis of myocardial infarction in women: a prospective cohort study

Anoop SV Shah,[1] Megan Griffiths,[1] Kuan Ken Lee,[1] David A McAllister,[2] Amanda L Hunter,[1] Anne Cruikshank,[3] Alan Reid,[3] Mary Stoddart,[4] Fiona Strachan,[1] Simon Walker,[4] Paul O Collinson,[5] Fred S Apple,[6] Alasdair J Gray,[7] Keith AA Fox,[1] David E Newby[1] and Nicholas L Mills[1]

[1]BHF Centre for Cardiovascular Science, University of Edinburgh, United Kingdom
[2]Centre for Population Health Sciences, University of Edinburgh, United Kingdom
[3]Department of Biochemistry, Southern General Hospital, United Kingdom
[4]Department of Biochemistry, Royal Infirmary of Edinburgh, United Kingdom
[5]Department of Cardiology, St George's Hospital and Medical School, United Kingdom
[6]Department of Laboratory Medicine and Pathology, University of Minnesota, United States of America
[7]Department of Emergency Medicine, Royal Infirmary of Edinburgh, United Kingdom

Correspondence and requests for reprints:

Dr Nicholas L Mills
BHF/University Centre for Cardiovascular Science
Chancellor's Building
University of Edinburgh
Edinburgh EH16 4SB
United Kingdom
Tel: +44 131 242 6515
Fax: +44 131 242 6379
E-mail: nick.mills@ed.ac.uk

Support: British Heart Foundation Special Project Grant (SP/12/10/29922), Clinical Research Scholarship (SS/CH/09/002), Intermediate Fellowship (FS/10/024/28266) and Chair Award (CH/09/002).

Table and Figures: 4

Table S1. Diagnosis of myocardial infarction using contemporary and high-sensitivity assays

	Contemporary assay [a]	High-sensitivity assay (single threshold) [b]	High-sensitivity assay (sex-specific threshold) [c]	P-value [d]	P-value [e]
**All patients** (n=1,126)					
**Type 1 myocardial infarction**	**172 (15%)**	**222 (20%)**	**242 (21%)**	**<0.001**	**0.003**
Type 2 myocardial infarction	47 (4%)	55 (5%)	56 (5%)	0.012	0.999
Myocardial injury	27 (2%)	38 (3%)	40 (4%)	<0.001	0.727
Unstable angina	38 (3%)	28 (2%)	28 (2%)	0.004	0.999
Other	842 (75%)	783 (70%)	760 (68%)	<0.001	0.004
**Men** (n=622)					
**Type 1 myocardial infarction**	**117 (19%)**	**142 (23%)**	**131 (21%)**	**0.001**	**0.002**
Type 2 myocardial infarction	27 (4%)	30 (5%)	28 (5%)	0.999	0.999
Myocardial injury	12 (2%)	15 (2%)	12 (2%)	0.999	0.999
Unstable angina	27 (4%)	20 (3%)	22 (4%)	0.074	0.480
Other	439 (71%)	415 (67%)	429 (69%)	0.035	0.035
**Women** (n=504)					
**Type 1 myocardial infarction**	**55 (11%)**	**80 (16%)**	**111 (22%)**	**<0.001**	**<0.001**
Type 2 myocardial infarction	20 (4%)	25 (5%)	28 (6%)	0.008	0.250
Myocardial injury	15 (3%)	23 (5%)	28 (6%)	<0.001	0.250
Unstable angina	11 (2%)	8 (2%)	6 (1%)	0.073	0.480
Other	403 (80%)	368 (73%)	331 (66%)	<0.001	<0.001

a. Contemporary assay single threshold 50 ng/L
b. High-sensitivity assay single threshold 26 ng/L
c. High-sensitivity assay sex-specific thresholds: women 16 ng/L and men 34 ng/L
d. McNemar’s test comparing diagnosis based on high-sensitivity troponin assay using sex-specific thresholds to the contemporary assay using a single threshold
e. McNemar’s test comparing diagnosis based on high-sensitivity troponin assay using sex-specific thresholds to the high-sensitivity assay using a single threshold

Table S2. Baseline characteristics of women with and without myocardial infarction where assays were concordant (groups 3 and 1 respectively) and those only identified using the high-sensitivity assay with sex-specific thresholds (group 2)*

	**Group 1** n=337	**Group 2** n=56	**Group 3** n=55
Age	65 (16)	76 (12)	75 (15)
Chest pain	264 (79)	42 (75)	48 (87)
**Risk Factors**			
Smoker	57 (19)	10 (20)	8 (15)
Hypertension	165 (49)	36 (64)	31 (56)
Hyperlipidaemia	135 (40)	28 (50)	20 (36)
Diabetes mellitus	43 (13)	11 (20)	9 (16)
**Past medical history**			
Ischaemic heart disease	124 (37)	34 (61)	17 (31)
Myocardial infarction	56 (17)	23 (41)	13 (24)
Ischaemic stroke	15 (4)	7 (12)	5 (9)
Transient ischaemic attack	21 (6)	1 (2)	3 (5)
Peripheral vascular disease	2 (1)	0 (0)	3 (5)
**Revascularisation**			
Previous PCI	32 (10)	7 (12)	7 (13)
Previous CABG	12 (4)	5 (9)	2 (4)
**Haemodynamics**			
Systolic pressure, mmHg	137 (25)	137 (30)	138 (30)
Diastolic pressure, mmHg	71 (14)	71 (17)	75 (18)
Heart rate, beats per minute	77 (18)	83 (21)	84 (18)
**Clinical chemistry**			
Haemoglobin, g/dL	130 (56)	127 (15)	125 (17)
Creatinine, mg/dL	72 (23)	90 (38)	95 (49)
Urea, mg/dL	6 (6)	9 (8)	7 (4)
Cholesterol, mmol/L	5 (1)	5 (1)	5 (1)
**Electrocardiography**			
ST elevation	9 (3)	5 (9)	16 (30)
ST depression	23 (7)	11 (20)	13 (24)
T wave inversion	63 (20)	15 (27)	12 (22)
**Admission drugs**			
Aspirin, n (%)	109 (33)	29 (54)	20 (36)
Clopidogrel, n (%)	37 (11)	9 (17)	4 (7)
B-blockers, n (%)	83 (25)	22 (41)	14 (25)
ACE inhibitors, n (%)	89 (27)	21 (39)	23 (42)
Statins, n (%)	123 (37)	30 (56)	20 (36)
Warfarin, n (%)	15 (5)	7 (13)	3 (5)
Proton pump inhibitor, n (%)	119 (36)	26 (48)	20 (36)

*Excludes patients adjudicated as type 2 myocardial infarction or myocardial injury

Table S3. Baseline characteristics of men with and without myocardial infarction where assays were concordant (groups 3 and 1 respectively) and those only identified using the high-sensitivity assay with sex-specific thresholds (group 2)*

	**Group 1** n=451	**Group 2** n=14	**Group 3** n=117
Age	61 (15)	64 (17)	67 (14)
Chest pain, n (%)	383 (85)	14 (100)	99 (85)
**Risk Factors**			
Smoker, n (%)	145 (34)	6 (46)	38 (34)
Hypertension, n (%)	227 (50)	7 (50)	61 (53)
Hyperlipidaemia, n (%)	210 (47)	9 (64)	54 (47)
Diabetes mellitus, n (%)	69 (15)	3 (21)	25 (22)
**Past medical history**			
Ischaemic heart disease, n (%)	193 (43)	9 (64)	54 (47)
Myocardial infarction, n (%)	135 (30)	8 (57)	33 (28)
Ischaemic stroke, n (%)	38 (8)	3 (21)	8 (7)
Transient ischaemic attack, n (%)	20 (4)	0 (0)	3 (3)
Peripheral vascular disease, n (%)	12 (3)	1 (7)	8 (7)
**Revascularisation**			
Previous PCI, n (%)	93 (21)	4 (29)	18 (16)
Previous CABG, n (%)	40 (9)	2 (14)	14 (12)
**Haemodynamics**			
Systolic pressure, mmHg	133 (22)	141 (23)	131 (22)
Diastolic pressure, mmHg	73 (15)	75 (14)	74 (14)
Heart rate, beats per minute	78 (18)	78 (15)	81 (22)
**Clinical chemistry**			
Haemoglobin, g/dL	141 (17)	142 (24)	138 (21)
Creatinine, mg/dL	82 (24)	133 (197)	104 (57)
Urea, mg/dL	6 (3)	7 (6)	7 (4)
Cholesterol, mmol/L	4 (1)	5 (1)	5 (1)
**Electrocardiography**			
ST elevation, n (%)	36 (8)	1 (8)	42 (36)
ST depression, n (%)	13 (3)	1 (8)	29 (25)
T wave inversion, n (%)	75 (17)	3 (23)	34 (29)
**Admission drugs**			
Aspirin, n (%)	178 (40)	9 (64)	46 (40)
Clopidogrel, n (%)	55 (12)	5 (36)	12 (11)
Beta-blockers, n (%)	119 (27)	8 (57)	34 (30)
ACE inhibitors / ARB, n (%)	149 (34)	5 (36)	38 (33)
Statin, n (%)	194 (44)	8 (57)	52 (45)
Warfarin, n (%)	28 (6)	1 (7)	6 (5)
Proton pump inhibitor, n (%)	139 (31)	6 (43)	31 (27)

*Excludes patients adjudicated as type 2 myocardial infarction or myocardial injury

Figure S1. Distribution of troponin and upper reference limits (99th percentile) in 4,590 samples from healthy men and women

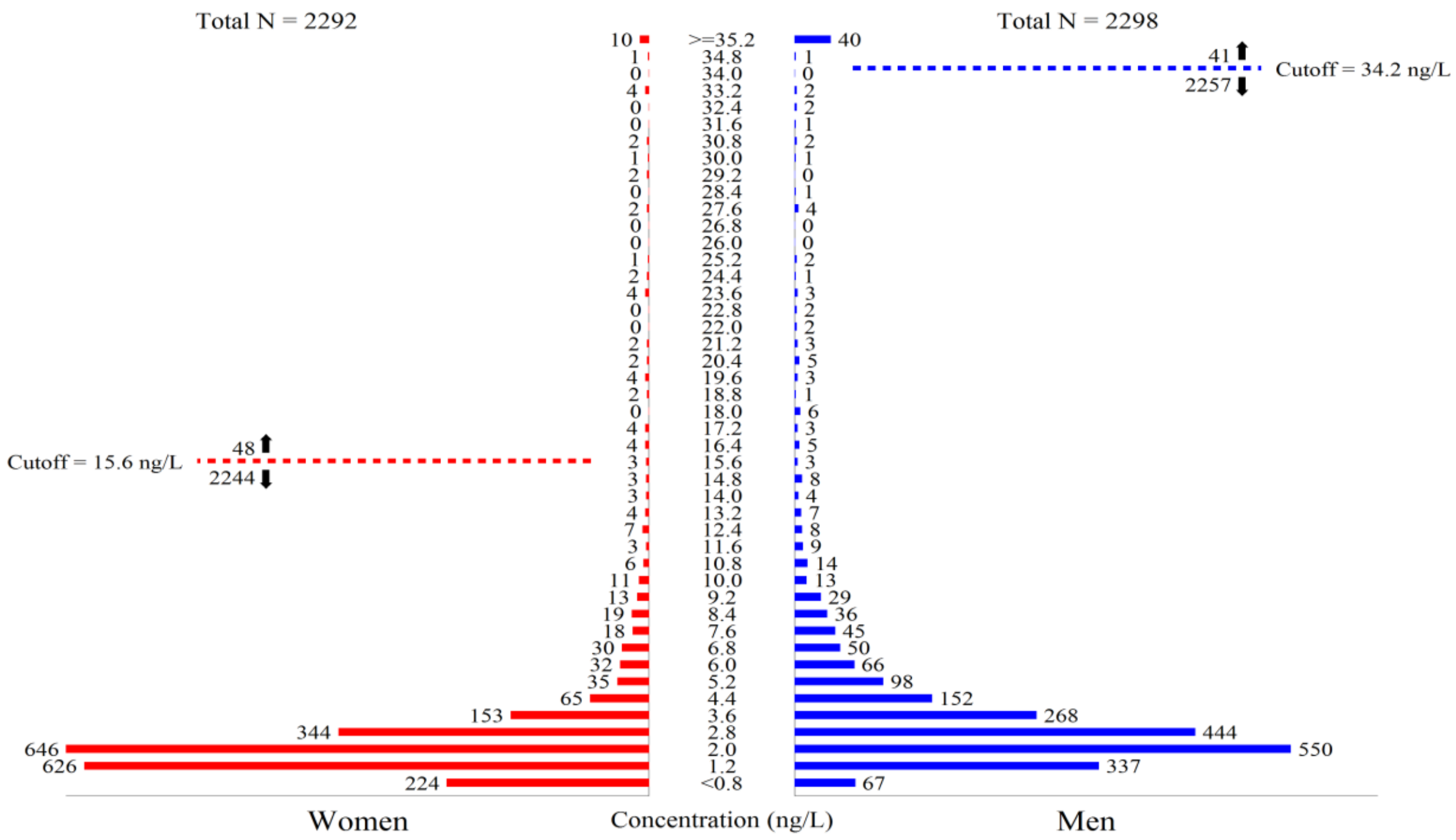

Reference population consists of 1,531 persons with an equal number of men and women. Inclusion criteria were age 21 to 75 years, no previous medical illness, normal renal function (eGFR >60 mL/min), glycated haemoglobin (HbA1c) ≤ 6% and normal B-type naturetic peptide concentrations (male ≤ 25 pg/mL and female ≤ 40 pg/mL) (Rogers et al *Clin Chem*; 2013 (10) A225).
